# Supplementary material for: Gut Microbiota-Derived Metabolites and Cardiovascular Disease Risk: A Systematic Review of Prospective Cohort Studies
Source: Nutrients. 2022 Jun 27;14(13):2654. doi: 10.3390/nu14132654 (PMC9268449; doi:10.3390/nu14132654)
Supplement: Supplementary file 1 [file nutrients-14-02654-s001.zip › nutrients-1750851-supplementary.pdf]

**Supplementary Files**

**Table S1**. PubMed and SCOPUS search strategies.

| **PubMed** |
|---|
| ((bile acids OR short-chain fatty acids OR branched-chain amino acids OR trimethylamine n-oxide OR TMAO OR cholic acid OR cholate OR chenodeoxycholic acid OR chenodeoxycholate OR taurocholic acid OR taurocholate OR glycocholic acid OR glycocholate OR taurochenodeoxycholic acid OR taurochenodeoxycholate OR glycochenodeoxycholic acid OR glycochenodeoxycholate OR glycocholenate_sulfate OR glycohyocholate OR glycolithocholate_sulfate OR glycoursodeoxycholate OR taurocholenate_sulfate OR taurodeoxycholate OR taurolithocholate_3_sulfate OR tauroursodeoxycholate OR deoxycholic acid OR deoxycholate OR lithocholic acid OR lithocholate OR acetic acid OR acetate OR propionic acid OR propionate OR butyric acid OR butyrate OR leucine OR isoleucine OR valine OR indole OR tryptophan OR indole-3-lactate OR indole-3-lactic acid OR indole-3-propionate OR indole-3-propionic acid OR indole-3-aldehyde OR indoxylsulphate OR indoxylsulfuric acid OR indole-3-acetate OR indole-3-acetic acid OR indole-3-carboxylate OR indole-3-carboxylic acid OR indole-acetaldehyde) AND (Cardiovascular Diseases) AND (Magnetic Resonance Spectroscopy OR High-Throughput Screening Assays OR Chromatography OR Mass Spectrometry) AND (humans[Filter]) AND (english[Filter]) NOT (review[PT]) NOT (systematic review[PT]) NOT (Randomized Controlled Trial [PT]) NOT (Clinical Trial [PT]) NOT (Meta-Analysis [PT])) |
| **Scopus** |
| TITLE-ABS ( ( ( bile AND acids ) OR ( short-chain AND fatty AND acids ) OR ( branched-chain AND amino AND acids ) OR ( trimethylamine AND n-oxide ) OR indole OR tryptophan ) AND ( cardiovascular AND diseases ) ) AND DOCTYPE ( ar ) AND LANGUAGE ( english ) |

**Table S2**. Quality assessment of the prospective cohort studies, based on the NEWCASTEL-OTTAWA criteria.[1]

| *Study* | *Representativeness of the Exposed Cohort* | *Selection of the Non-Exposed Cohort* | *Ascertainment of Exposure* | *Demonstration That Outcome of Interest Was Not Present at Start of Study* | *Comparability of Cohorts on the Basis of the Design or Analysis* | *Assessment of Outcome* | *Was Follow-Up Long Enough for Outcomes to Occur* | *Adequacy of Follow Up of Cohorts* |
|---|---|---|---|---|---|---|---|---|
| *Lee, Y.* | * | * | * | * | ** | * | * | * |
| *Frazier, R.* | * | * | * | * | ** | * | * | * |
| *Amrein, M.* | * | * | * | * | ** | * | * | * |
| *Chen, Y.Y.* | * | * | * | * | ** | * | | |
| *Fretts, A.M.* | * | * | * | * | ** | * | * | * |
| *Winther, S.A.* | * | * | * | * | ** | * | * | * |
| *Ringel, C.* | * | * | * | * | ** | * | * | * |
| *Israr, M.Z.* | * | * | * | * | ** | * | * | * |
| *Croyal, M.* | * | * | * | * | ** | * | * | * |
| *Roncal, C.* | * | * | * | * | ** | * | * | * |
| *Yu, B.* | * | * | * | * | ** | * | * | * |
| *Wang, C.* | * | * | * | * | ** | * | * | * |
| *Cheng, S.* | * | * | * | * | ** | * | * | * |
| *Tobias, D.K.* | * | * | * | * | ** | * | * | * |
| *Alonso, A.* | * | * | * | * | ** | * | * | * |
| *Fan, P.C.* | * | * | * | * | ** | * | * | * |

| | | | | | | | | |
|---|---|---|---|---|---|---|---|---|
| *Huang, J.* | * | * | * | * | ** | * | * | * |
| *Balasubramanian, R.* | * | * | * | * | ** | * | * | * |
| *Du, X.* | * | * | * | * | ** | * | * | * |

[1] A study can be awarded a maximum of one star for each numbered item within the Selection and Outcome categories. A maximum of two stars can be given for Comparability.

**Table S3**. Quality assesment of the nested case-control studies, based on the NEWCASTEL-OTTAWA criteria.[1]

| ***Study*** | ***Is the case definition adequate?*** | ***Representativeness of the cases*** | ***Selection of Controls*** | ***Definition of Controls*** | ***Comparability of cases and controls on the basis of the design or analysis*** | ***Ascertainment of exposure*** | ***Same method of ascertainment for cases and controls*** | ***Non-Response rate*** |
|---|---|---|---|---|---|---|---|---|
| *Liu, J.* | * | * | * | * | ** | * | * | * |
| *Papandreou, C.* | * | * | * | * | ** | * | * | * |

[1] A study can be awarded a maximum of one star for each numbered item within the Selection and Exposure categories. A maximum
of two stars can be given for Comparability.
